# Supplementary material for: MetaRibo-Seq measures translation in microbiomes
Source: Nat Commun. 2020 Jun 29;11:3268. doi: 10.1038/s41467-020-17081-z (PMC7324362; doi:10.1038/s41467-020-17081-z)
Supplement: Supplementary file 10 — Supplementary Data 7 [file 41467_2020_17081_MOESM10_ESM.zip › File2/Confidence_High_Taxonomy/84527_out.krona.html]

Javascript must be enabled to view this page.

members
magnitude
magnitudeUnassigned
count
unassigned
taxon
rank

84527\_out

50

2
50
superkingdom

phylum
50
976

50
200643
class

50
171549
order

family
815
1

1
816
genus

species

SRS078176\_contig\_number\_6186
47678
1

family
171552
49

genus
838
49

165179
41
species

SRS013521\_contig\_number\_2396SRS013638\_contig\_number\_1683SRS013687\_contig\_number\_4529SRS014415\_contig\_number\_1109SRS015794\_contig\_number\_7798SRS016541\_contig\_number\_12890SRS017307\_contig\_number\_27861SRS019285\_contig\_number\_341SRS023914\_contig\_number\_1282SRS043667\_contig\_number\_5678SRS043841\_contig\_number\_8959SRS043841\_contig\_number\_10157SRS044535\_contig\_number\_contig-100\_16579.94641SRS045713\_contig\_number\_21841SRS046502\_contig\_number\_10943SRS047433\_contig\_number\_19294SRS049995\_contig\_number\_33137SRS050752\_contig\_number\_22843SRS053356\_contig\_number\_33463SRS053398\_contig\_number\_25237SRS058145\_contig\_number\_contig-100\_6634.41237SRS064757\_contig\_number\_14169SRS065397\_contig\_number\_contig-100\_2738.93785SRS074586\_contig\_number\_1388SRS077194\_contig\_number\_4327SRS077991\_contig\_number\_12SRS097958\_contig\_number\_contig-100\_3784.116171SRS104036\_contig\_number\_7830SRS1041031\_contig\_number\_24651SRS1041134\_contig\_number\_contig-100\_2856.70001SRS1041140\_contig\_number\_16301SRS104327\_contig\_number\_7674SRS143181\_contig\_number\_124SRS144297\_contig\_number\_contig-100\_3521.93930SRS144506\_contig\_number\_54070SRS144537\_contig\_number\_contig-100\_1521.262245SRS144603\_contig\_number\_contig-100\_1825.70928SRS144714\_contig\_number\_1197SRS146764\_contig\_number\_28441SRS147614\_contig\_number\_4714SRS148253\_contig\_number\_contig-100\_1549.97957

159272
5

SRS018888\_contig\_number\_contig-100\_4238.43323SRS019910\_contig\_number\_contig-100\_634.140908SRS044535\_contig\_number\_387SRS077641\_contig\_number\_contig-100\_3716.119371SRS104912\_contig\_number\_contig-100\_24052.24053
species

3
1263102
species

SRS045195\_contig\_number\_contig-100\_33270.33271SRS054059\_contig\_number\_contig-100\_4650.4651SRS103987\_contig\_number\_24428
